# IQ Domain GTPase-Activating Protein 1 is Involved in Shear Stress-Induced Progenitor-Derived Endothelial Cell Alignment

**DOI:** 10.1371/journal.pone.0079919

**Published:** 2013-11-22

**Authors:** Lila Rami, Patrick Auguste, Noélie B. Thebaud, Reine Bareille, Richard Daculsi, Jean Ripoche, Laurence Bordenave

**Affiliations:** 1 Bioingénierie Tissulaire, Université de Bordeaux, U 1026, F-33000 Bordeaux, France; 2 Bioingénierie Tissulaire, U1026, INSERM, Bordeaux, France; 3 LAMC, UMR 1029, Université Bordeaux, Talence, France; 4 LAMC, UMR 1029, INSERM, Talence, France; 5 CIC-IT BioDiMI, CHU de Bordeaux, Bordeaux, France; University of Illinois at Chicago, United States of America

## Abstract

Shear stress is one of mechanical constraints which are exerted by blood flow on endothelial cells (ECs). To adapt to shear stress, ECs align in the direction of flow through adherens junction (AJ) remodeling. However, mechanisms regulating ECs alignment under shear stress are poorly understood. The scaffold protein IQ domain GTPase activating protein 1 (IQGAP1) is a scaffold protein which couples cell signaling to the actin and microtubule cytoskeletons and is involved in cell migration and adhesion. IQGAP1 also plays a role in AJ organization in epithelial cells. In this study, we investigated the potential IQGAP1 involvement in the endothelial cells alignment under shear stress. Progenitor-derived endothelial cells (PDECs), transfected (or not) with IQGAP1 small interfering RNA, were exposed to a laminar shear stress (1.2 N/m^2^) and AJ proteins (VE-cadherin and β-catenin) and IQGAP1 were labeled by immunofluorescence. We show that IQGAP1 is essential for ECs alignment under shear stress. We studied the role of IQGAP1 in AJs remodeling of PDECs exposed to shear stress by studying cell localization and IQGAP1 interactions with VE-cadherin and β-catenin by immunofluorescence and Proximity Ligation Assays. In static conditions, IQGAP1 interacts with VE-cadherin but not with β-catenin at the cell membrane. Under shear stress, IQGAP1 lost its interaction from VE-cadherin to β-catenin. This “switch” was concomitant with the loss of β-catenin/VE-cadherin interaction at the cell membrane. This work shows that IQGAP1 is essential to ECs alignment under shear stress and that AJ remodeling represents one of the mechanisms involved. These results provide a new approach to understand ECs alignment under to shear stress.

## Introduction

Endothelial cells (ECs) are continuously exposed *in vivo* to mechanical stress (tangential fluid shear stress, cyclic circumferential strain and blood pressure). When exposed to fluid shear stress, mature ECs and ECs derived from endothelial progenitor cells become elongated and aligned in the direction of flow, which involves AJ reshaping [1], [2]. ECs alignment is associated to a translocation of the vascular endothelial cadherin (VE-cadherin) and β-catenin, inducing the disruption of the AJ [3]. ECs sense mechanical stimuli and convert them to biochemical signals leading to the activation of signalling pathways that are partly elucidated [4]–[6]. The mechanosensory and mechanotransducer complex of shear stress at cell-cell junction consists of PECAM-1, vascular endothelial growth factor receptor-2 (VEGFR-2) and VE-cadherin [5], which functions mainly as an adapter system. Moreover, shear stress stimulates the formation of the VEGFR-2/VE-cadherin/β-catenin complex to activate MAP kinases pathway [7]. The same protein complex is formed after VEGF stimulation and implies IQGAP1 [8].

IQGAP1 is a large modular protein (189 kDa) that displays multiple partnerships (for review: [9]) and mediates numerous aspects of mammalian biology by binding to and regulating the function of numerous interacting proteins. More particularly, IQGAP1 acts as a scaffold protein in coupling cell signaling to the actin and microtubule cytoskeletons in cell migration and adhesion [10]. The interactions of IQGAP1 with the AJ components control epithelial cell-cell adhesion. However, the consequences of IQGAP1 knockdown are less clear. Depending on the cell type, IQGAP1 can stabilize or destabilize the AJ through its interaction with E-cadherin and/or β-catenin and implicating Rac1 as partner [11], [12]. For example, in Madin Darby canine kidney II (MDCKII) cells, TPA induces AJ destabilization by decreasing the Rac1-GTP active form and, consequently, by decreasing the Rac1-IQGAP1 complex. IQGAP1 can bind β-catenin which dissociates from α-catenin inducing AJ destabilization [11]. In PANC-1 cells, IQGAP1 is constitutively associated with the E-cadherin complex, stabilizing cell-cell junctions. During PANC-1 cells stimulation, Rac1-GTP increases, binds IQGAP1 which dissociates from the E-cadherin complex inducing AJ destabilization [12]. In the culminant of the nonmetazoan Dictyostelium discoideum β- and α-catenin recruit IQGAP1 to lateral epithelial cell-cell contact but β- and α-catenin localization at cell-cell contact is independent of IQGAP1 [13]. In human ECs, IQGAP1 colocalized and interacts with VE-cadherin and β-catenin at sites of cell-cell contact and knockdown of IQGAP1 with siRNA inhibits the localization of VE-cadherin at AJ [8] and disrupts the endothelial barrier [14]. The role of IQGAP1 in EC morphological changes, elongation and alignment, under shear stress and in AJ remodeling induced by shear stress has not been extensively studied in details and only for a short period of shear stress [15].

As far as shear stress partially disassembles AJ in ECs [1], we investigated whether IQGAP1 was involved in AJ under shear stress conditions in human progenitor derived endothelial cells (PDECs). We used IQGAP1 siRNA and studied PDECs morphological changes under shear stress. We detected the interactions that IQGAP1 established with the VE-cadherin and β-catenin in situ at the cell membrane, at different times of exposure to a shear stress of arterial type (1.2 N/m^2^) by Proximity Ligation Assays (PLA).

## Materials and Methods

### Ethics Statement

Human umbilical cord blood was provided by the Etablissement Français du Sang (EFS), and the use of the cord blood for research was approved in an agreement between EFS and INSERM. All donors provided written informed consent to EFS.

### Isolation, Expansion of Human Cells Derived from Mononuclear Cell (MNC) Cultures and PDECs Characterization

PDECs were isolated, cultured and characterized as previously described by Thébaud *et al*. [16]. Briefly, MNCs were isolated from 45 mL of buffy coat obtained from human umbilical cord blood from healthy donors after dilution with phosphate-buffered saline (PBS) and 2 mM Ethylenediaminetetraacetic acid (EDTA) by density gradient centrifugation in 1.077 g/mL Histopaque® solution (Sigma-Aldrich), washed several times with PBS and cultured in EGM-2 (endothelial cell growth medium-2, Lonza-Verviers, France) with supplements from the kit and 5% foetal calf serum (FCS) (GIBCO Life Technologies, Karlsruhe, Germany), on collagen-coated well plates (collagen type I, rat Tail, BD Biosciences). A total of 10^7^ cells/well were seeded on a 24 culture-well plate. At day 4, non-adherent cells were removed from cultures of MNCs. Cells were fed with fresh medium every second day. Colonies with cobblestone-like morphology appearing after 2–3 weeks in culture were selected and cells were expanded over several passages using standard cell cultures procedures [17].

The stability of the endothelial phenotype during the expansion of these cells has been investigated on passages 5 and 15. The following criteria used for characterization were cellular uptake of Ac-LDL binding of UEA-1 lectin, immunofluorescent stainings for CD31, VE-cadherin and von Willebrand factor (vWF), flow cytometric analysis of CD45, CD34, CD31 and VEGFR-2, and Matrigel tube formation assay as previously described by Thébaud et al. [16].

### Application of Shear Stress

For shear stress experiments, a parallel plate culture flow chamber was used as described in [18]. Briefly, PDECs were seeded on a 7.5×3.8 cm glass slide coated with type 1 collagen (10,000 cells/cm^2^). Four days after seeding, PDECs formed a confluent monolayer and were subjected to a laminar shear stress at 1.2 N/m^2^ for 4 h, 8 h, 30 h and 48 h, in Iscove’s Modified Dulbeco’s Medium (IMDM), 5% FCS at 37°C. PDECs unsubmitted to shear stress cultured in IMDM 5% FCS served as static controls. Cell orientation was determined by measuring angle between cells axis and the direction of flow using the “angle tool” in Image J.

### Immunofluorescence Staining

Cells exposed or not to a laminar shear stress were washed once with phosphate buffer saline (PBS), fixed with 4% paraformaldehyde (PFA) for 20 min at room temperature and washed three times in PBS. After permeabilization with 0.1% triton X100 for 1 min, the samples were incubated for 15 min with 1% Bovine Serum Albumin (BSA) (w/v) and 1% FCS (v/v) in PBS at room temperature. Then in order to show proteins localization, cells were incubated 2 h at room temperature with a mouse (Upstate, 1/100) or rabbit (Santa Cruz, 1/100) antibody to IQGAP1 and one of the following antibodies: a mouse (BD Pharmingen; 1/100) or rabbit (Cell signaling; 1/400) antibodies to VE-cadherin or a rabbit polyclonal antibody to human β-catenin (Cell Signaling, 1/100). After three washes, cells were incubated for 1 hour at room temperature with the appropriate secondary antibody labeled with Alexa488 or Alexa568 (Molecular Probes (Invitrogen); 1/200). Cells were mounted under cover slips with ProLong containing DAPI to label nuclei (Invitrogen - Molecular Probes) and observed using TCS SP5 confocal microscope (Leica, Wetzlar, Germany). After selection of cell membrane area, fluorescence intensities were quantified by using the threshold function of the Image J software.

### Cell Transfection with siRNAs

The corresponding sequences for the siRNAs were as follows: siIQGAP1 672: 5′-UGAAGCUAUUGACCGUAGAdTdT-3′ and siLUC: 5′-CGUACGCGGAAUCUUCGAdTdT-3′ as a negative control (MWG Biotech). Gene-specific siRNA oligomers (80 pmole) were diluted in 1 mL Opti-MEM, reduced serum medium (Opti-MEM, Invitrogen), mixed with 15 µL of lipofectamine (RNAiMax, Invitrogen) according to the manufacturer’s protocol. After 20 min of incubation at room temperature in a type I collagen pretreated dish (10 cm^2^), PDECs in suspension were added to the mix (1.10^5^ cells/cm^2^). Adherent cells were transfected again 24 h after the first transfection. 48 h after the first transfection, cells were trypsinized and seeded on flow plates pretreated with type I collagen (80,000 cells/cm^2^). Finally, cells were exposed to shear stress 72 h after the first transfection. The PDECs transfected with a non-target control siRNA (siLUC) were used as controls and the quality of transfection and the siRNA efficacity were evaluated by western blot and immunofluorescence staining.

Cell viability after transfection was evaluated by an in vitro Toxicology Assay Kit based on Sulforhodamine B (SIGMA Aldrich) according to the manufacture’s protocol. Briefly, cells transfected with siLUC or siIQGAP1 and exposed (48 h) or not (control) to shear stress were fixed by gently layering of cold 50% (w/v) Trichloro Acetic Acid (TCA) solution on top of the growth medium (¼ volume) for 1 h at 4°C. After several washes with water, glass slides were air dried and then covered with 0.4% Sulforhodamine B solution for 30 min at room temperature. Dye in excess was removed by washing rapidly with 1% (vol/vol) Acetic Acid and the incorporated dye is finally solubilized in 10 mM Tris base solution and transferred in microplates for optical density determination at 565 nm using a microplate reader (Victor™ X3– PerkinElmer).

### Permeability Assay

96 h after cell transfection (siLuc or siIQGAP1 672 sequences), permeability assay was assessed by measuring the passage of FITC-labeled dextran (Molecular weight: 70 kDa, Invitrogen®) across a Transwell® (Millipore) seeded with not transfected PDECs or PDECs transfected with the LUC or IQGAP1 siRNA. Cells were starved during 1 hour (medium replacement was performed in wells and Transwells®). Then, 200 mL of serum free medium containing 1 mg/mL of FITC-labeled dextran or 1 mg/mL of non-labeled dextran (negative control) were added in the Transwell®. 200 mL of serum free medium containing 1 mg/mL of FITC-Dextran was added to uncellularized Transwell® as positive control. At 15, 45 and 180 min, 100 mL of each sample were transferred in a 96 wells plate for fluorescence measurement (Victor™ X3– PerkinElmer).

### Western Blot

Cells exposed (4 h, 8 h, 30 h and 48 h) or not (control) to shear stress were lysed for 45 min at 4°C in lysis buffer containing 10 mM Tris-HCl pH 7.7, 2 mM EDTA, 0.15 M NaCl, 1%NP40 and 5 mM β-mercaptoethanol. Control cells were lysed 72 h, 96 h and 120 h after the first transfection. The samples were cleared by centrifugation (20 min at 16,000 g) and the supernatant was assayed for protein concentration (BCA assay, Thermo Fisher Scientific). Proteins (20 µg) were resolved by sodium dodecylsulfate-polyacrylamide gel electrophoresis (SDS-PAGE, 10% acrylamide) and transferred to a polyvinylidene difluoride membrane (Immobilon P, Millipore). Membranes were blocked for 1 h in Tris-buffered saline-Tween (TBS-T: 20 mM, Tris-HCl, pH 7.4, 150 mM NaCl, 0,05% Tween 20) containing 5% nonfat milk. Then, blots were probed with the rabbit polyclonal anti-IGAP1 antibody (1∶3000), or the mouse monoclonal anti-VE-cadherin antibody (1∶1000) or the rabbit polyclonal anti-β-catenin antibody (1∶1000) overnight at 4°C. After washing, the secondary peroxidase-labeled goat anti-rabbit antibody (1∶15,000) or goat anti-mouse antibody (1∶10000) was applied for 1 h at room temperature. Immunoreactive detection was performed by chemiluminescence (ECL Plus Western Blotting Chemiluminescent Substrate, GE Healthcare) and the signal intensity was quantified using Image J software.

### Rac1 Activation Assay

Rac1 proteins are activated very rapidly and transiently. To assess the maximal activation rate, Rac1 activity was assayed for cells exposed 30 minutes to shear stress after adjustments. For measurements of Rac1 activity, the appropriate G-LISA™ activation Assay Biochem Kit™ (Cytoskeleton) was used according to manufacturer’s instructions. Briefly, PDECs were grown to sub-confluence and exposed to shear stress (1.2 N/m^2^) for 30 min; cells not sheared were used as control. After washing with ice-cold PBS, ice-cold cell lysis buffer was added, and cell lysates were harvested by centrifugation at 15,000 *g* at 4°C for 2 min. An aliquot was kept on ice for protein concentration measurement, and samples were snap frozen at −70°C. After adjustment of protein concentration, cell lysates were thawed and 50 µl of lysate were added to the wells of the RAC-GTPase binding plate, which is coated with a Rac-GTP binding domain (p21 binding domain, PBD). Additional wells were filled with lysis buffer or nonhydrolyzable Rac as a negative or positive control, respectively. 50 µL of Rac1 primary antibody (1∶200 in antibody dilution buffer) were added and incubated for 45 min on an orbital microplate shaker (400 rpm, RT) after what, wells were washed and incubated with 50 µL of secondary anti-horseradish peroxidase (HRP)-labeled antibody (1∶100) on the orbital microplate shaker (45 min, RT). Then, 50 µL of horseradish peroxidase (HRP) detection reagent were added and incubated at 37°C for 15 min. HRP stop buffer (50 µ0l) was added, and the colorimetric signal was measured immediately at 490 nm using a microplate reader spectrophotometer (Victor™ X3– PerkinElmer). Before each incubation, the plate was washed three times with PBS.

### In situ Proximity Ligation Assay (PLA)

The Duolink™ in situ PLA allows the detection and the quantification of IQGAP1 interactions with the AJ proteins VE-cadherin and β-catenin. Briefly, PDECs exposed or not (control) to flow were rinsed with PBS, fixed with 4% PFA for 20 min at room temperature, permeabilized with 0.1% Triton X100 for 1 min and incubated in blocking buffer (1% BSA (w/v), 1% FCS (v/v) in PBS) for 15 min. Cells were immunolabeled with the same primary antibodies as those described above. Then, PDECs were incubated for 2 h at 37°C with secondary antibodies with attached PLA probes, supplied in the Duolink kit. The subsequent procedure was the same as that described in the manufacturer’s instruction. Finally, cells were mounted under a cover slip with ProLong containing DAPI (Invitrogen - Molecular Probes) and images were taken and analyzed using the TCS SPE confocal microscope and LASAF software (Leica, Wetzlar, Germany). The red fluorescence signal indicated that the two proteins were separated by less than 30–40 nm [19]. To quantify protein interactions labeled by PLA, images were analyzed using ImageJ. The raw images were opened and converted into 8-bit file and then converted to binary images. The number of red dots at the cell membrane was calculated by “analyse particules” in image J. A minimum of 20 cells per conditions were analyzed.

### Statistical Analyis

Data were expressed as mean +/− SEM. Statistical analysis was performed by the One-way analysis of variance (ANOVA) followed by the post hoc Turkey test (Prism 5, GraphPad).

## Results

### Shear Stress Induces PDECs Alignment in the Direction of Flow

**Figure 1:**
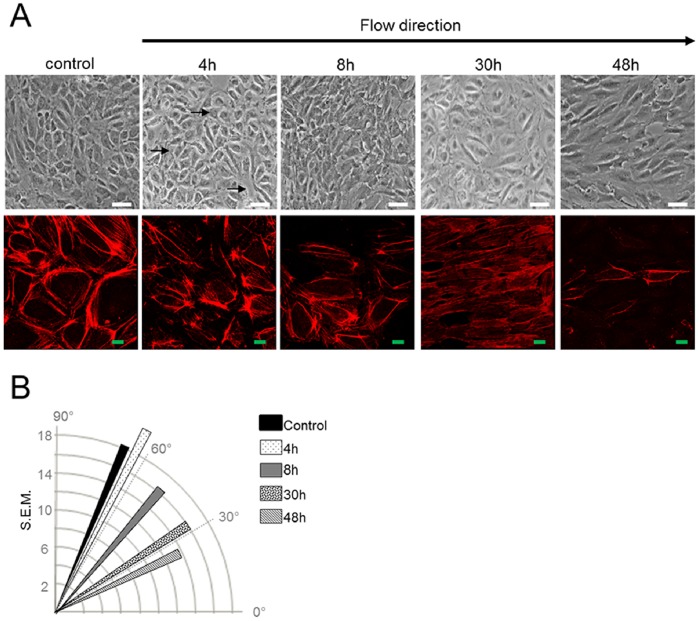
PDECs align in the direction of flow under shear stress. (A) PDECs exposed to static or shear stress conditions (1.2 N/m^2^) were observed after fixation by optical microscopy. Cells not exposed to flow were used as control. Progressively, PDECs organized in a monolayer of contiguous cells, detached each other (arrows at 4 h) and aligned in the direction of flow (Scale bar: 100 µm). In lower panel, the actin cytoskeleton was labeled in PDECs exposed or not to shear stress, showing cortical actin and stress fiber formation within cells. (B) Cells direction has been quantified under static and shear stress conditions using the ImageJ software. The vertical axis and circular arcs corresponding to the standard error of mean (S.E.M.) and the horizontal axis representing the flow direction.

In this study, PDECs were subjected to a laminar shear stress of 1.2 N/m^2^ corresponding to an arterial blood flow. In static conditions PDECs kept their typical cobblestone morphology (fig. 1A). Under shear stress conditions, cells elongated and became orientated with the shear vector. Time-course experiments indicated that the process of alignment of a confluent PDECs layer was a slow process; after 4 h of flow, cells dissociated from each other being only linked by some cell-cell contacts (fig. 1A) and complete cell alignment in the direction of flow was obtained at 48 h as shown by microscopy and quantitative analysis of angles (fig. 1A and B). The cortical actin present in static cultures tended to disappear under shear stress, giving way to stress fiber distributed parallel to the direction of flow at 30 hours (fig. 1A).

### IQGAP1 Silencing Impairs PDECs Alignment Under Shear Stress

**Figure 2:**
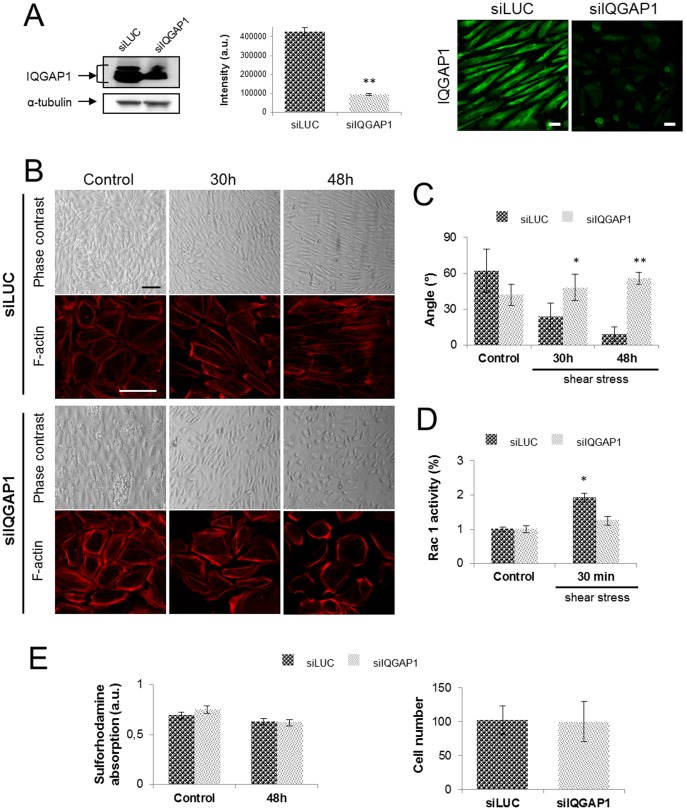
IQGAP1 is necessary for cell-cell adhesion and PDECs alignment. The IQGAP1 extinction rate has been evaluated by immunofluorescence staining of IQGAP1 and western blot before (not shown) and after 48 h of exposition to shear stress (scale bar: 10 µm) (A). PDECs were transfected with siRNA against IQGAP1 (siIQGAP1) or Luciferase (siLUC, control), exposed to shear stress for the indicated times (scale bar: 100 µm) and F-actin was stained with coupled-TRITC phalloïdine antibody (scale bar: 100 µm) (B). Angles of siLUC or siIQGAP1 transfected PDECs orientation under shear stress have been measured for 3 independent experiments and are represented in (C). The Rac1 activity has been quantified for PDECs transfected with siLUC or siIQGAP1 and maintained in static conditions or exposed to shear stress for 30 h and 48 h, showing the non Rac1 activation for siIQGAP1-transfected PDECs (D). Cell viability has been checked by Sulforhodamine test and cell number has been quantified in static conditions (control) and after 48 h of shear stress (E).

In order to investigate the implication of IQGAP1 in the alignment process induced by shear stress, PDECs, in which IQGAP1 expression was down-regulated, were exposed to shear stress for 30 h and 48 h. First, we analyzed IQGAP1 protein in cells treated with siIQGAP1 by immunofluorescence staining and by Western blot (fig. 2A). A siRNA targeted luciferase (siLUC) was used as control. After 48 h of shear stress, IQGAP1 western blot quantification showed a 75% downregulation in cells transfected with siIQGAP1 as compared with PDECs transfected with siLUC (fig. 2A) indicating that the protein downregulation was maintained all along shear stress exposition until 48 h (control and 30 h of shear stress not shown). PDECs transfected with siLUC were aligned in the direction of flow after 48 h of shear stress (fig. 2B and C). On the opposite, in PDECs transfected with siIQGAP1 a reduction of cell-cell contacts between 30 h and 48 h and an inhibition of cell alignment were observed (fig. 2B and C).

To adapt to local mechanical load and subsequent injury caused by the shear stress, ECs aligned in the direction of flow induced thanks to the actin cytoskeleton remodeling. Transfected PDECs (siLuc or siIQGAP1) exhibit cortical actin under static conditions (fig. 2B). At 48 h, stress fibers were observed in the direction of flow, within siLUC-transfected PDECs. Cortical actin but no stress fibers were observed in siIQGAP1-transfected cells after 48 h of shear stress (fig. 2B). It is well known that actin remodeling induced by shear stress is dependent on Rac1 activity in the first 30 min of shear [5], [20]–[22]. To investigate the Rac1 participation in the PDECs alignment, the Rac1 activity has been assayed for cells exposed or not to shear stress (1.2 N/m^2^) with or without IQGAP1 siRNA. The assay shows a significant increase of Rac1 activity in cells transfected by siLUC and exposed to shear stress (30 min) in comparison with the control cells, not exposed to shear stress (fig. 2D). In contrast, the Rac1 activity remains constant for cells transfected with the siIQGAP1 exposed or not to shear stress (fig. 2D). These results mean that the shear stress Rac1 activation, essential for cell alignment needs IQGAP1.

The latter result could not be attributed to a toxicity of transfection since viability of siIQGAP1 transfected cells was not affected after 48 h of flow (fig. 2E), as measured by a sulforhodamine assay and the cell number were not affected after 48 h of flow (fig. 2E).

### Shear Stress Induces Adherens Junction Remodeling and IQGAP1 Delocalization

**Figure 3:**
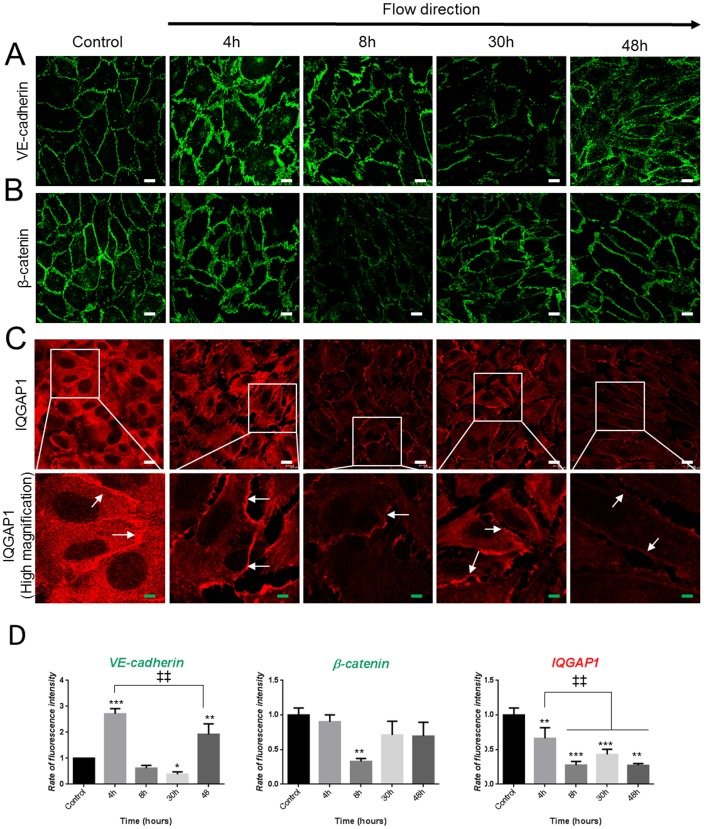
Shear stress induces the adherens junction remodeling (A and B) and IQGAP1 delocalization (C). VE-cadherin, β-catenin and IQGAP1 immunofluorescence staining in control conditions and after flow are represented in A, B and C respectively. Fluorescence intensities at the cell membrane are quantified in (D) and total protein expression were assessed by western blot for cells maintained in static conditions (control) or exposed to shear stress for 8 h (E). (*p<0.05; **p<0.01; ***p<0.001 refer to comparison with control conditions. ^‡‡^p<0.01 refer to indicated comparisons) (white scale bar: 10 µm; green scale bar: 3 µm).

Under shear stress, PDECs alignment is allowed by the loss of the cell-cell adhesion [23]. Using immunofluorescence staining and quantitative analysis of fluorescence intensity, we followed localization and quantification of two AJ proteins, VE-cadherin and β-catenin. In static conditions (control), VE-cadherin and β-catenin were expressed at the cell membrane (fig. 3A and B). At 4 h, the membrane labeling was discontinuous for both AJ proteins (fig. 3A and B) forming “dashes” as described by Noria et al. 1999 [1]. The localization of VE-cadherin and β-catenin became punctuate and sparse after 30 h and 8 h of shear stress, respectively (fig. 3A and B). After 48 h of shear stress, PDECs alignment was concomitant with a re-established continuous cell membrane expression of the two AJ proteins as observed with porcine endothelial cells [1].

**Figure 4:**
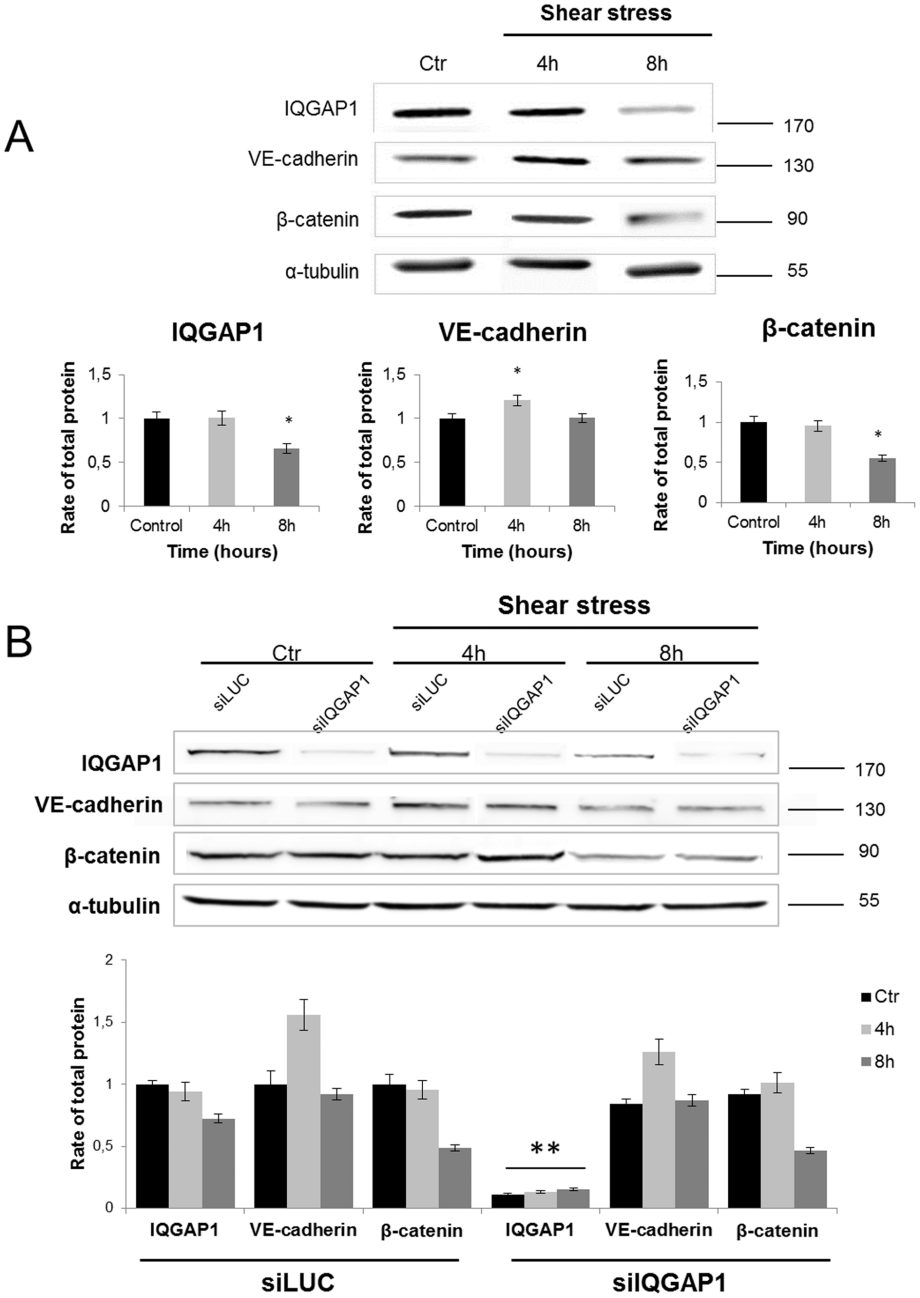
Total protein expression of IQGAP1, VE-cadherin and b-catenin in PDECs transfected or not with siRNA, before and after shear stress exposure. The total expression of IQGA1, VE-cadherin and β-catenin in non-transfected PDECs was investigated by western blot in static conditions (Ctr) and after 4 h and 8 h of shear stress, and quantified in comparison with the static condition (Ctr) (*p<0.05) (A). The same experiment was performed on siRNA (LUC or IQGAP1) transfected cells (**p<0.01 refer to comparison with siLUC conditions) (B).

During this kinetics, quantitative expression of fluorescence intensity showed that VE-cadherin labeling is significantly increased by 4 h of shear stress as compared to static control (fig. 3D) and corroborated by western blot (fig. 4A); the labeling decreased at 8 h and 30 h and increased at 48 h (fig. 3D). The β-catenin expression follows a different kinetics than VE-cadherin. Fluorescence intensity did not change at 4 h, was transiently lowered at 8 h (p<0.01) as observed on western blot analysis (fig. 3D and 4A) before recovering at 30 h and beyond (fig. 3D). Taken together, immunofluorescence staining and western blot quantification assume the adherence junction remodeling under shear stress.

IQGAP1 expression and localization were studied by immunofluorescence. Before PDECs exposition to flow, IQGAP1 was expressed in cytoplasm and at the cell-cell contacts (fig. 3C, control). From early times of shear stress (4 h and 8 h), IQGAP1 was less expressed in cytoplasm, but a discontinuous strong expression was maintained at the cell membrane, until 48 h (fig. 3C, magnification, arrows). IQGAP1 fluorescent intensity quantitation at the cell membrane showed that IQGAP1 expression decreased from 8 h (p<0.001) and remained low at 48 h (p<0.01). Western blot shows in figure 4A that protein level is lowered after 8 h. The total protein expression of VE-cadherin, β-catenin are not modified by the siLUC or siIQGAP1 transfection and IQGAP1 expression is not modified by siLUC transfection (fig. 4B).

### IQGAP1 Interactions with VE-cadherin or β-catenin are Modified by Shear Stress

**Figure 5:**
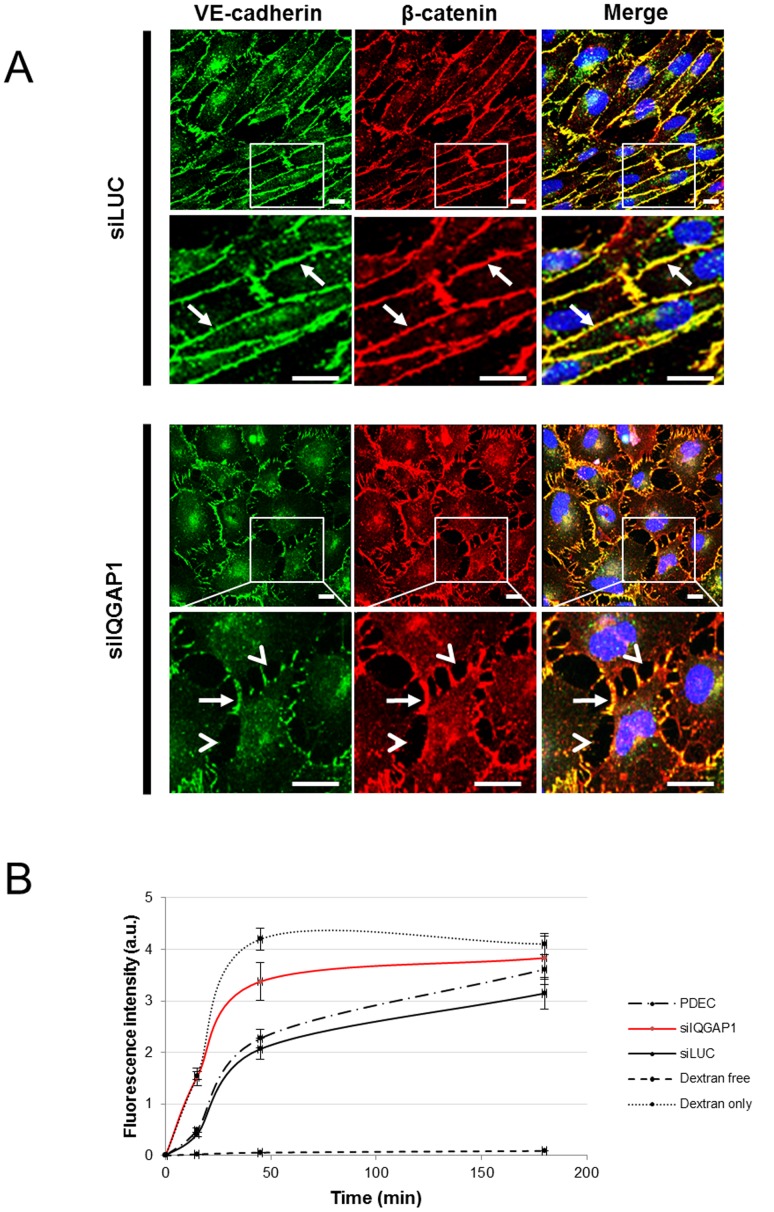
IQGAP1 downregulation induces the lack of cell cohesiveness. In (A) are represented immunofluorescence staining of VE-cadherin and β-catenin in PDECs transfected with siIQGAP1 or siLUC, under static conditions. VE-cadherin and β-catenin expression were discontinuous at the cell membrane in siIQGAP1-transfected PDECs (arrows). Arrowheads show lacunas formed by the adherens junction weakness (Scale bar = 100 µm). Permeability assay was performed by quantification of FITC-dextran (70 kDa) passage through a monolayer of not transfected or siRNA (LUC or IQGAP1) -transfected PDECs seeded in a Transwell®. The transfection with siIQGAP1 seems to increase the monolayer permeability (B). *PDECs*: untransfected cells; *siIQGAP1*: IQGAP1 siRNA-transfected cells; *siLUC*: Luciferase siRNA-transfected cells; *Dextran free*: serum free medium without dextran (negative control); *Dextran only*: dextran-FITC added to serum free medium without cells.

Since David et al. 2011 [14] have shown in microvascular brain endothelial cells the involvement of IQGAP1 in cell cohesiveness, we compared in our PDECs model, the cell cohesiveness by immunofluoresence staining of AJ proteins in PDECs transfected with siIQGAP1 or siLUC (fig. 5A, arrows). In static conditions, after IQGAP1 down-regulation, VE-cadherin appeared weakened at the cell membrane, punctuate and sparse compared with siLUC. Similar observations were obtained for β-catenin (fig. 5A); cells were less cohesive, interactions between cells were not continuous, and lacunas were observed between adjacent cells (fig. 5A, arrowheads). Using a Dextran-FITC solution, we noticed that the permeability of the confluent endothelial cobblestone in siIQGAP1 transfection conditions was increased in comparison with siLUC transfection condition (fig. 5B).

**Figure 6:**
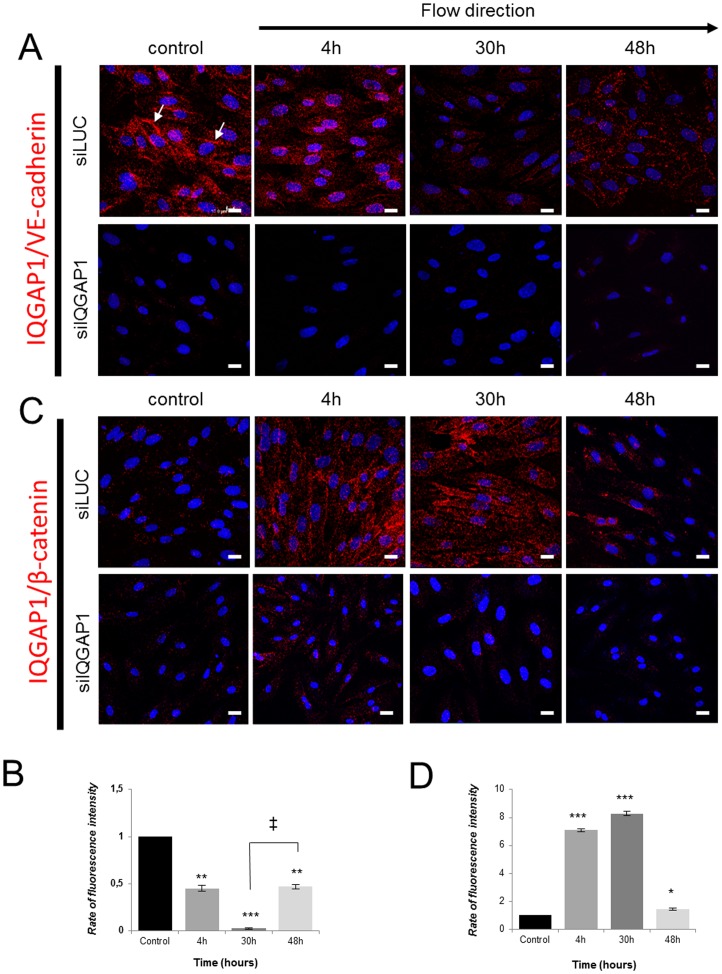
IQGAP1 and adherens junction proteins interactions are modulated under shear conditions. PDECs are exposed to shear stress and interactions between IQGAP1 and VE-cadherin (A) and IQGAP1 and β-catenin (B) were analyzed with the Duolink™ kit. Proteins interactions at cell membrane are showed by arrows. (B) and (D) show the rate of fluorescence intensity (normalized to that of control) at the cell membrane, after shear stress for IQGAP1/VE-cadherin and IQGAP1/β-catenin interactions, respectively. (*p<0.05; **p<0.01; ***p<0.001 refer to comparison with control conditions. ^‡^p<0.05 refers to indicated comparisons) (Scale bar: 10 µm).

To investigate the potential role of IQGAP1 in AJ, interactions between IQGAP1 and VE-cadherin or β-catenin within cells were studied by *in situ* Proximity Ligation Assays (PLA). In this technique a red spot reflects that the two proteins are separated by less than 30–40 nm, nearby making interaction. The specificity of the PLA was controlled in PDECs in which IQGAP1 expression was down-regulated by siRNA; no interactions could be detected between IQGAP1 and VE-cadherin or β-catenin (fig. 6A and C, lower panels).

In static conditions, as expected [8], IQGAP1 interacts with VE-cadherin at cell-cell contacts, and a decrease in these interactions was observed under shear stress (fig. 6A, upper panel). This experiment suggests an interaction between IQGAP1 and VE-cadherin under static conditions; this interaction was inhibited under shear stress (4 h and 30 h). When cells were aligned in direction of the flow (48 h) VE-cadherin and IQGAP1 interacted again at the cell membrane (fig. 6A, upper panel and 6B). In static condition, only some interactions between IQGAP1 and β-catenin were detected (fig. 6C, upper panel). Under shear stress, IQGAP1/β-catenin interactions increased significantly (7 to 8 times at 4 h and 30 h of shear stress) and fall down at 48 h when cells are aligned (fig. 6C, upper panel and 6D). These results may suggest that at early time of flow, IQGAP1 interactions switches from VE-cadherin to β-catenin, allowing cell alignment.

**Figure 7:**
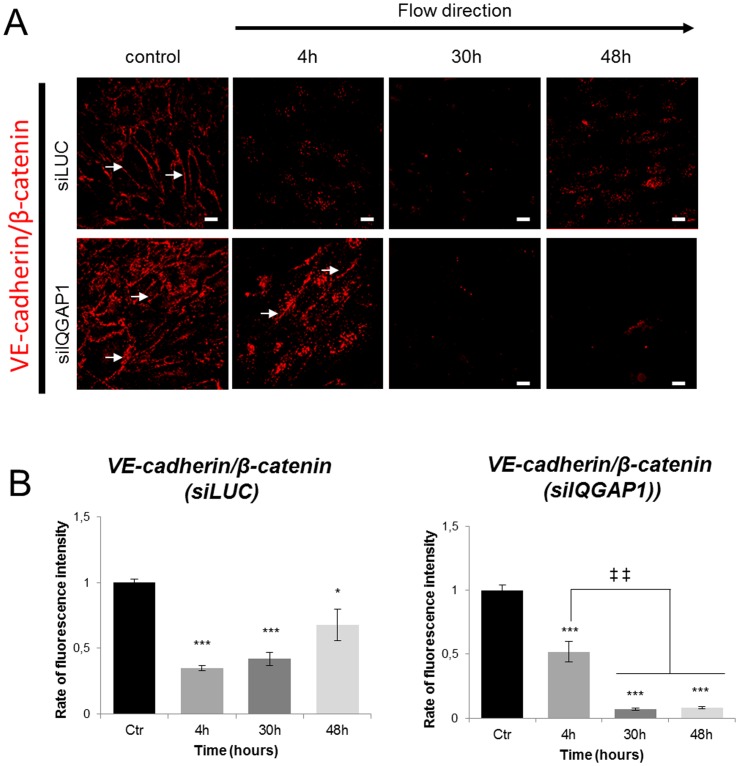
VE-cadherin and β-catenin interactions are modulated under shear conditions. PDECs are exposed to shear stress and interactions between VE-cadherin and β-catenin, were labeled with the Duolink kit in cells transfected with either siLUC or siIQGAP1 (A). Proteins interactions at cell membrane are showed by arrows. B and C show the rate of fluorescence intensity (normalized to that of control) after shear stress for VE-cadherin/β-catenin interaction for siLUC or siIQGAP1 transfected cells, respectively. (*p<0.05; **p<0.01; ***p<0.001 refer to comparison with control conditions. ^‡^p<0.05 refers to indicated comparisons) (Scale bar: 10 µm).

To investigate whether this switch could be responsible of the disruption of the AJ, we studied the interaction between VE-cadherin and β-catenin by PLA in PDECs transfected with siLUC or siIQGAP1. In static condition, VE-cadherin and β-catenin interacted in control cells or in cells transfected with siIQGAP1 (fig. 7A control panels). This interaction at the cell membrane significantly decreased under shear stress (fig. 7A upper panel and 7B left panel) and seems to reappear at 48 h in the intracellular area when cells were aligned. In contrast, in IQGAP1-depleted PDECs, the number of interactions between the two proteins decreased but interactions were still present at 4H, and completely disappeared at 30 h and 48 h (fig. 7A lower panel and 7B right panel). All these results suggest that the VE-cadherin/β-catenin interaction loss is delayed in IQGAP1 siRNA-transfected PDECs (fig. 7).

## Discussion

IQGAP1 under shear stress conditions has only been studied in short time course experiments (20 min) and at a low flow rate (0,1 N/m^2^) [15]. Here we studied the role of IQGAP1 in the morphological adaptation of PDECs submitted to long period of arterial shear stress. PDECs have the capacity to proliferate and migrate [16]. Several studies have shown their therapeutic potential in tissue ischemia, repair of blood vessels and bioengineering of prosthetic grafts. Therefore, it is important to understand how these cells adapt under shear stress conditions. Little is known about the mechanisms governing the behavior of PDECs under shear stress. The present study is the first one, to our knowledge, to investigate IQGAP1 participation to cell alignment under arterial shear stress in endothelial cells and more precisely PDECs.

Mechanical forces from blood flow, exert strong regulatory influences on the physiology and pathology of the cardiovascular system [24]. As expected from results obtained with endothelial progenitor cells from peripheral blood [2], PDECs from cord blood aligned in the direction of flow. In our experiences confluent cells were used, which may explain the delayed acquisition of this phenotype, as the degree of confluency is a critical parameter in the kinetics of cell alignment under shear stress [25].

In a first part we provide evidence that in PDECs, IQGAP1 is involved in shear stress PDECs alignment since its down regulation impairs the cell-cell cohesion. Cell alignment is closely related to adherens junction remodeling involving VE-cadherin and β-catenin. IQGAP1 is known to anchor adherens junction by maintaining VE-cadherin - β-catenin binding at cell membrane [26]. Thus, we investigated the adherens junction remodeling focusing on VE-cadherin, β-catenin and IQGAP1 expression kinetics all along shear stress exposure. The observed delocalization of the VE-cadherin and the β-catenin is a process that probably started at the onset of flow exposure since labeling appears modified from 4 h, whereas protein levels are not modified. The latter findings are in favor of a delocalization. After 8 h, our data suggest that VE-cadherin and β-catenin degradation occurs, on the basis of fluorescence intensity variation at the cell membrane and results of western blot analysis. After 48 h, proteins are relocalized and cells are aligned (Fig. 2 A and B), which is consistent with Noria *et al*. results [1]. Thus, the cell membrane expression of junctional proteins VE-cadherin and β-catenin decreases at the onset of shear stress exposure, and increases when cells are aligned in the direction of flow after 48 h.

The IQGAP1 protein has numerous protein partners via its interaction domains and is involved in many cellular processes including cell adhesion and migration [27], [28]. The role of IGPAP1 on the stability of adherens junctions appears to be cell type-dependent. Our results suggest that IQGAP1 is important in maintaining endothelial cell alignment under shear stress conditions through its control on the actin remodeling and the adherens junction stability. Cell alignment is depended on the actin remodeling through the Rac1 activation, in the first 30 min of exposition to shear stress [5], [20]–[22]. IQGAP1-downregulated cells exhibit cortical actin in static conditions that remains up to 48 h of flow. This non-alignment is induced by the lack of stress fibers formation, which is the direct consequence of the non-activation of Rac1 without IQGAP1. The Rac1 activity measured in Figure 2D for siIQGAP1 - transfected PDECs may be related to the IQGAP1 residue. Nevertheless, the remaining IQGAP1 amount does not seem to be enough to induce the Rac1 activation under shear stress. IQGAP1 may play a role of mediator between shear stress and cell alignment. In literature, the role of IQGAP1 on adherens junction stability is contradictory, probably because it is cell specific. IQGAP1 destabilizes the adherens junction by its interaction with β-catenin, preventing it from interacting with E-cadherin and α-catenin in fibroblasts on Madin-Darby canine kidney II cells [11], [29]. In pancreatic carcinoma cells, another group showed that the binding of IQGAP1 to the β-catenin promotes cell-cell cohesion by stabilizing cell adhesion junction [12]. In EC, IQGAP1 is implicated in stabilizing adherens junctions in static conditions, downstream of Rac1 and Cdc42 [27]. In our study, the shear stress induces the disruption of the adherent junction: indeed, VE-cadherin/β-catenin dissociation is observed at the membrane after 4 h. Almost 75% of the basal level of interaction between VE-cadherin and β-catenin is reached after 48 h, but remains low when IQGAP1 is down-regulated. These results suggest the essential role of IQGAP1 in the stabilization of the adherence junction under flow. All experiments suggest that IQGAP1 is important for the adherent junction stability in static conditions and under shear stress when cells are aligned.

After 4 h, we observed the loss of IQGAP1/VE-cadherin interaction, the increased IQGAP1/β-catenin interaction and a delay in the VE-cadherin/β-catenin dissociation at the cell membrane suggesting that in static condition IQGAP1 interaction with VE-cadherin stabilizes the adherent junction, shear stress induces IQGAP1/β-catenin interaction and concomitant loss of VE-cadherin/IQGAP1 and VE-cadherin/β-catenin interactions. In pancreatic carcinoma cells, IQGAP1 interaction with β-catenin induces the dissociation of VE-cadherin and β-catenin, leading to the adherent junction weakness [12]. It has also been reported that such a shift in IQGAP1 interaction between VE-cadherin and β-catenin can occur in human brain micro-vascular cells of the brain, with, as a result, a destabilization of the EC barrier [30]. These results may suggest a potential role of IQGAP1 for the adherent junction remodeling at the onset of shear stress.

In conclusion, PDECs alignment under shear stress is dependent on the reorganization of the AJ and stress fibers network. VE-cadherin plays an important role in this phenomenon. IQGAP1 could play a role in both processes through its role in the AJ integrity and the actin cytoskeleton organization [25]. In summary we suggest that IQGAP1 is involved in the alignment induced by shear stress and that one underlying mechanism is related to its role in the AJ organization.
